# RNAi-Based Biocontrol of Wheat Nematodes Using Natural Poly-Component Biostimulants

**DOI:** 10.3389/fpls.2019.00483

**Published:** 2019-04-17

**Authors:** Konstantin B. Blyuss, Farzad Fatehi, Victoria A. Tsygankova, Liudmyla O. Biliavska, Galyna O. Iutynska, Alla I. Yemets, Yaroslav B. Blume

**Affiliations:** ^1^Department of Mathematics, University of Sussex, Brighton, United Kingdom; ^2^Department of Chemistry of Bioactive Nitrogen-Containing Heterocyclic Compounds, Institute of Bioorganic Chemistry and Petrochemistry, National Academy of Sciences of Ukraine, Kyiv, Ukraine; ^3^Department of General and Soil Microbiology, Zabolotny Institute of Microbiology and Virology, National Academy of Sciences of Ukraine, Kyiv, Ukraine; ^4^Department of Cell Biology and Biotechnology, Institute of Food Biotechnology and Genomics, National Academy of Sciences of Ukraine, Kyiv, Ukraine; ^5^Department of Genomics and Molecular Biotechnology, Institute of Food Biotechnology and Genomics, National Academy of Sciences of Ukraine, Kyiv, Ukraine

**Keywords:** crop protection against nematodes, streptomycete-derived biostimulants, RNA interference, wheat (*Triticum aestivum* L.), cereal cyst nematode (*Heterodera avenae*)

## Abstract

With the growing global demands on sustainable food production, one of the biggest challenges to agriculture is associated with crop losses due to parasitic nematodes. While chemical pesticides have been quite successful in crop protection and mitigation of damage from parasites, their potential harm to humans and environment, as well as the emergence of nematode resistance, have necessitated the development of viable alternatives to chemical pesticides. One of the most promising and targeted approaches to biocontrol of parasitic nematodes in crops is that of RNA interference (RNAi). In this study we explore the possibility of using biostimulants obtained from metabolites of soil streptomycetes to protect wheat (*Triticum aestivum* L.) against the cereal cyst nematode *Heterodera avenae* by means of inducing RNAi in wheat plants. Theoretical models of uptake of organic compounds by plants, and within-plant RNAi dynamics, have provided us with useful insights regarding the choice of routes for delivery of RNAi-inducing *biostimulants* into plants. We then conducted *in planta* experiments with several streptomycete-derived biostimulants, which have demonstrated the efficiency of these biostimulants at improving plant growth and development, as well as in providing resistance against the cereal cyst nematode. Using dot blot hybridization we demonstrate that biostimulants trigger a significant increase of the production in plant cells of si/miRNA complementary with plant and nematode mRNA. Wheat germ cell-free experiments show that these si/miRNAs are indeed very effective at silencing the translation of nematode mRNA having complementary sequences, thus reducing the level of nematode infestation and improving plant resistance to nematodes. Thus, we conclude that natural biostimulants produced from metabolites of soil streptomycetes provide an effective tool for biocontrol of wheat nematode.

## 1. Introduction

One of the biggest challenges to agriculture comes from pests and parasites that cause enormous economic losses and threaten global food security. Plant parasitic nematodes are known to infest almost all cultivated crops, and they result in losses of over 20% of the annual yield (Jung and Wyss, 1999), and estimated economic losses of over $130 billion worldwide (Chitwood, 2003). The largest damage to crops can be attributed to sedentary endoparasitic nematodes of the *Tylenchoidea* superfamily, which includes root-knot nematodes (*Meloidogyne* spp.) and cyst nematodes (*Globodera* and *Heterodera* spp.) (Tamilarasan and Rajam, 2013). Symptoms of nematode-related plant disease include wilting, stunting, and enhanced susceptibility to other diseases. While cyst nematodes are quite specific in the type of host plants they choose to parasitize on, root-knot nematodes do not have such high host specificity and can infect a very wide range of different crops. Irrespective of the type of nematodes, when their juveniles reach the second stage J2 (infective juveniles), they migrate from the soil into new host roots, or use the same host plant as the one used by their parent. In case of migration through the soil, these juveniles use lipids stored in their gut to sustain them for a short period of time needed to get established in a new host. Inside the plant, the nematodes migrate intra-cellularly (cyst nematodes) or inter-cellularly (root-knot nematode) to become sedentary in the roots and form feeding cells that support further development and reproduction (Bird and Kaloshian, 2003; Caillaud et al., 2008). These feeding cells later transform into either syncytia in the case of cyst nematodes, or multinucleate giant cells for root-knot nematodes, which subsequently result in the formation of galls and plant death after several cycles of nematode reproduction. In the case of wheat (*Triticum aestivum* L.), its major nematode parasites are cereal cyst nematode, primarily *Heterodera avenae* (Nicol et al., 2007; Peng et al., 2009), and root lesion nematode *Pratylenchus* (*P. neglectus* and *P. thornei*) (Vanstone et al., 1998). Smiley and Nicol (2009) and Nicol and Rivoal (2008) provide nice overviews of different species of wheat nematodes, including the discussion of their epidemiology, as well as approaches to management.

Over the years various control measures have been proposed to reduce the negative impact of nematodes on the performance of crops in general, and wheat in particular. These include application of chemical nematicides, development and use of organic cultivars, and crop rotation (Tamilarasan and Rajam, 2013). In the case of wheat, management and sanitation can be achieved using rotation with non-cereal, resistant or tolerant cultivars (Smiley and Nicol, 2009). Despite successes of synthetic chemical pesticides in mitigating damage and protecting crop from pests, other options are currently being explored due to the emergence of resistance in pest populations, and concerns over safety to humans and environment associated with ground water contamination and residues in food (Thomason, 1987).

One promising approach for crop protection and control of nematodes is *RNA interference* (RNAi) (Saurabh et al., 2014; Kamthan et al., 2015; Li et al., 2015; Kanakala and Ghanim, 2016; Rehman et al., 2016; Ali et al., 2017a; Banerjee et al., 2017; Borel, 2017; Majumdar et al., 2017), which is a fundamental biological process, through which eukaryotic cells are able to post-transcriptionally control expression of specific genes. The process starts with either exogenous (in the case of plant viruses) or endogenous (host's own) double-stranded RNA (dsRNA), which is cleaved by the Dicer enzyme into 21–25nt long small interfering RNA (siRNA). These are then unwound, and the passenger strand is discarded, while the guide strand is loaded onto Argonaute protein to form the RNA-induced silencing complex (RISC) that is able to degrade complementary mRNA, thus stopping it from being translated into protein (Hammond et al., 2000; Filipowicz, 2005; Carthew and Sontheimer, 2009). Host cells can also take RNAs from viruses and make them double stranded using RNA-dependent RNA polymerases (Wassenegger and Pelissier, 1998; Lipardi et al., 2001; Makeev and Bamford, 2002). One should also note that sRNAs do not only target mRNAs. In fact, most siRNAs target non-coding RNAs (Hüttenhofer et al., 2005; Chen and Aravin, 2015; Wang et al., 2015), while miRNA target mRNAs, as well as non-coding RNAs, which results in the production of phasiRNA (Ye et al., 2014; Liu et al., 2015). For the purpose of controlling plant infections, RNAi can be used in two different ways: it can protect the plants from infection, and it can be used to target the parasites. Protecting plants from infection relies on the plant possessing appropriate dsRNA, so that it could produce the RISC, which would stop expression of proteins necessary for the successful infection. Targeting parasites is a strategy where upon feeding on plants, insects or nematodes would consume dsRNA that upon entering their gut would trigger the process of RNAi against their own genes, thus reducing their fecundity and causing mortality (Fairbairn et al., 2007; Charlton et al., 2010; Dalzell et al., 2010; Li et al., 2010, 2015; Duan et al., 2012). In both of these approaches the important first step is supplying plants with the appropriate dsRNA. This can be achieved either by developing transgenic plants able to produce necessary dsRNA through their own cell machinery (Zhang et al., 2015), or by providing it externally through spraying, root soaking etc. (Li et al., 2015; Joga et al., 2016; Heidebrecht, 2017). It has been shown recently that it is possible to develop transgenic plants carrying RNAi constructs providing protection against fungal infections of peanut (Arias et al., 2015) and wheat (Chen et al., 2016). One of the biggest challenges with spraying dsRNA on crops on agricultural scale is that it gets washed away with rain and is quite quickly degraded by the soil. A recent work has demonstrated that this problem can be overcome by loading dsRNA on clay nano-particles that in a field experiment stayed on the surface of leaves up to 30 days after application of the spray (Mitter et al., 2017).

In the context of nematode infections of crops, transgenic plants can quite effectively produce dsRNA that targets various nematode housekeeping genes, as well as parasitism or effector genes. For example, tobacco plant has been designed to express dsRNA of target genes of *Meloidogyne incognita* (Yadav et al., 2006), soybean has been engineered to express dsRNA to target a few house-keeping genes of *Heterodera glycines* (Klink et al., 2009; Li et al., 2010), and rapeseed plant has been designed to express dsRNA to target an important secretory protein of *Heterodera schachtii* (Tsygankova et al., 2013). Feeding dsRNA directly to nematode larvae is able to induce RNA silencing in western corn rootworm (Bolognesi et al., 2012) and colorado potato beetle (Baum et al., 2007). Urwin et al. (2002) have shown how octopamine can induce uptake of dsRNA by *H. glycines* and *Globodera pallida*.

Another prominent alternative strategy for crop protection is the use of biopesticides, in particular, natural biostimulants that are usually metabolites of plants or soil bacteria (Chandler et al., 2011; Ruiu, 2015; Yakhin et al., 2017). A major source of natural biostimulants are soil actinobacteria, such as *Saccharopolyspora* and *Streptomyces* genera. One example is *Streptomyces avermitilis* that produces *Avermectins* having insecticidal and nematicidal properties (Putter et al., 1981; Turner and Schaeffer, 1989). These macrocyclic lactone compounds target the GABA receptors in the peripheral nervous system of the insects and nematodes, causing inhibition of neurotransmission and paralysis of the neuromuscular systems (Bloomquist, 1996; Cabrera et al., 2013). Products of different streptomycetes have recently been shown provide effective nematicidal action (Ruanpanun et al., 2010; Rashad et al., 2015; Kaur et al., 2016). Recent work has shown that metabolites produced by some of the streptomycetes have a significant positive effect on plant performance through improved growth and development, and they also yield protection against nematode through stimulating production in plant cells of appropriate si/miRNA (Jailani and Mukherjee, 2017; Tsygankova et al., 2019). More specifically, metabolites and cell culture supernatant of soil streptomycetes *S. avermitilis* (Biliavska et al., 2012; Iutynska, 2012), *S. netropsis* (Biliavska et al., 2016a), and *S. violaceus* (Biliavska et al., 2016b) have been shown to possess strong antagonistic activity against various phytopathogenic micromycetes and bacteria (Iutynska et al., 2017). Earlier work (Biliavska et al., 2012, 2015a; Iutynska et al., 2017) has shown that when the selected streptomycetes are cultivated in liquid nutrient media, they simultaneously produce a number of distinct biologically active compounds, such as antibiotics (heptaene antibiotic candidin for *S. netropsis*, anthracycline antibiotics rhodilunantsin A and rhodilunantsin B for *S. violaceus*, and macrocyclic avermectins for *S. avermitilis*), amino acids, lipids, phytohormones (auxins, cytokinins, gibberellins, abscisic acid), and steroid compounds (cholesterol, ergosterol, sitosterol, stigmasterol, 24-epibrassinolide, squalene). The presence of these different biological products in the cultural liquid supernatant and biomass ethanol extracts means that the resulting biostimulants are actually complex poly-component products, which together provide a significant improvement of plant growth and development (Ponomarenko and Iutynska, 2011). This positive effect of biostimulants on plant growth has been demonstrated through improved callogenesis and organogenesis in wheat (Tsygankova et al., 2016, 2017). Biostimulants developed on the basis of metabolites of these streptomycetes have also shown nematicidal effects on root-knot nematode *M. incognita in vitro* (Biliavska et al., 2015a), as well as bioprotective RNAi-mediated effects against cyst nematode *H. schachtii* in rape (Tsygankova et al., 2013, 2014a), *Brassica rapa* subs. pekinensis (Chinese cabbage) (Biliavska et al., 2016c), and sugar beet (Tsygankova et al., 2012b), as well as on both of these nematodes in sugar beet and cucumber (Tsygankova et al., 2014b). It should be noted that we are using the term “biostimulant” loosely to describe poly-component substances without discriminating them by the mode of their action, such as biopesticides, plant growth regulators etc., hence avoiding the need to abide by one of the more restrictive definitions Calvo et al., 2014; Brown and Saa, 2015; du Jardin, 2015; Nardi et al., 2016.

To facilitate the development of optimal strategies for delivery of biostimulants into the plants, it is essential to understand how complex mixtures of chemical compounds are taken up by plants from the soil, as well as how they are subsequently transported within plants. Experimentally driven mathematical models suggest that equilibrium concentrations of chemical compounds in plants are reached very quickly, when plants are taking up compound from the soil through their roots (Briggs et al., 1983; Fryer and Collins, 2003; Rein et al., 2011). Theoretical models of RNAi (Groenenboom and Hogeweg, 2008; Neofytou et al., 2016a,b, 2017) have shown that the rate at which proliferating cells of meristematic tissue mature into healthy plant cells plays an important role in determining the success of RNAi-based plant response, which suggests that the transport of these products to different parts of the plant during its growth has a knock-on effect on their availability for consumption by nematodes. Furthermore, one should be mindful of the fact that in the case of poly-component mixtures, diffusion and uptake of different chemical components and their subsequent within-plant transport can significantly differ due to their various characteristics, such as lipophilicity, acidity, and electrical charge. Existing studies on the dynamics of germination and associated water uptake suggest that a significant role in this process is played by the seed morphology, which should be important in the case where biostimulants are applied directly to germinating seeds prior to them being sowed, rather than to the soil.

This paper analyses how poly-component biostimulants obtained on the basis of metabolites of soil streptomycetes can be used to protect wheat (*Triticum aestivum* L. cv. Zimoyarka) against the cereal cyst nematode *H. avenae* through stimulating production in plant cells of si/miRNA that are complementary with nematode mRNA upon being consumed by nematode from plants. The underlying idea of this methodology is the so-called *host-induced gene silencing* (HIGS), whereby the transfer of RNA in the form of either dsRNA or si/miRNA generated within host results in silencing essential nematode genes that are involved in nematode multiplication and/or egg production, parasitism and housekeeping (Fairbairn et al., 2007; Lilley et al., 2012; Nunes and Dean, 2012; Koch and Kogel, 2014). A very important point is that biostimulants used in this study are natural products of soil streptomycetes that are able to deliver targeted protection against the nematode, thus providing a safe and effective tool of biocontrol.

One particular problem for efficient plant-induced RNAi is a significant variability that has been observed in the efficiency of RNAi in nematodes in response to differences in sRNA concentrations, sizes of dsRNA constructs, and the duration of exposure (Gheysen and Vanholme, 2007; Maule et al., 2011; Lilley et al., 2012). Another major challenge is identifying specific RNAi compounds that are being transferred between plants and nematodes, i.e., whether it is the unprocessed dsRNA that subsequently has to be processed into siRNA by nematodes, or the siRNA, resulting from processing of these dsRNAs by plants using their DICER enzymes (Gheysen and Vanholme, 2007; Lilley et al., 2012). Even though significant amount of work has been done recently on trying to understand trans-kingdom transfer of small RNAs, and more specifically on the transfer of RNA between plants and their parasitic nematodes, many aspects of such transfer remain poorly understood (Knip et al., 2014; Sarkies and Miska, 2014; Han and Luan, 2015; Zhou et al., 2017; Cai et al., 2018; Baldrich et al., 2019), thus limiting our ability to design effective tools for host-induced RNAi.

Efficiency of plant-induced RNAi relies on the knowledge of appropriate nematode genetic targets for RNAi, and some progress has been made recently on identifying such targets for a number of agriculturally important nematodes (Koch and Kogel, 2014). In one recent study, Liu et al. (2016) have shown the effectiveness of RNAi on silencing the expression of *HaEXPB2* in *H. avenae* by *in vitro* soaking *H. avenae* larvae in homologous dsRNA, thus reducing its parasitism on *N. benthamiana*. Yang et al. (2017) have sequenced mRNA from both pre- and post-parasitic stages of *H. avenae*, providing an extensive characterization of development, metabolism, and parasitism genes. A recent work by Cui et al. (2018) has identified three specific *H. avenae* genes *c*59821_*g*1, *c*69968_*g*1, and *c*45915_*g*1 associated with lethal phenotypes, which suggests that they can be used as effective genetic targets for plant-induced RNAi. Similarly, Zheng et al. (2015) have studied transcriptome of second-stage juveniles of *H. avenae* and shown that silencing unigene38116, unigene102492, and unigene38007 genes, which are homologs of *C. elegans* genes *C*18*D*1.3, *F*38*E*11.7, and *C*06*G*4.2, respectively, by soaking nematode larvae in the gene-specific siRNA has lethal effect on the nematode. Kumar et al. (2014) have also analyzed transcriptome of second-stage juveniles of *H. avenae*, showing that it has the highest degree of similarity to a potato cyst nematode *G. pallida* and identified a number of candidate genes that can be used as targets for RNAi.

As mentioned earlier, one of the biggest challenges to RNAi-based biocontrol is the production in plants of si/miRNA complementary to nematode mRNA, as well as the delivery of necessary RNAi products to nematodes. Using some of the above-mentioned insights from mathematical models of plant uptake of chemical compounds, as well as the dynamics of RNAi in plants, we perform experiments on triggering nematode-specific si/miRNA production in wheat by using several streptomycete-derived biostimulants. The results suggest that these biostimulants are indeed very effective in protecting wheat against the cereal cyst nematode.

## 2. Materials and Methods

### 2.1. Biostimulants and Experimental Set-Up

Biostimulants used in this study are products based on metabolites of soil streptomycetes *S. avermitilis, S. netropsis*, and *S. violaceus*. Avermectins produced by *S. avermitilis* have long been known to possess insectidical, acaricidal, and nematicidal properties (Putter et al., 1981; Cabrera et al., 2013). In light of these observations, a strain *S. avermitilis* IMV Ac-5015 was grown in liquid organic soya media in bioreactors for 7 days at a temperature of +28±1°C (Biliavska et al., 2012, 2015b; Iutynska et al., 2017). After reaching the phase of stationary growth, the streptomycete's biomass was separated by centrifugation. Extraction of the streptomycete's metabolites in the biomass and supernatant of the cultural liquid was then carried out with ethanol at a ratio of 1:1 or 1:3 for one day, and subsequently the biomass was centrifuged. The biostimulant denoted as *Avercom* was obtained as a mixture of this cultural liquid supernatant extract and biomass extract at a 1:1 ratio, and was then stored at +4°C. Chemical analysis of this biostimulant showed that it contains avermectins at concentration of 100 μg/mL. The second biostimulant, *Avercom nova-2*, is a derivative of Avercom and contains 50mL of cell culture supernatant added to 50 mL of biomass extract of *S. avermitilis* IMV Ac-5015, with the addition of 0.01 mM of chitosan, a natural compound with known elicitor effects in plants (Hadrami et al., 2010; Malerba and Cerana, 2016). Exactly the same methodology was used to produce *Phytovit* from the streptomycete *S. netropsis* IMV Ac-5025, and *Violar* from *S. violaceus* IMV Ac-5027, with the only distinction being that for Phytovit and Violar the ratio of cultural liquid supernatant extract to biomass extract was 4:1. All these biolostimulants were tested in the laboratory setting prior to being used for plant treatment.

To investigate the content of auxins, cytokinins, and abscisic acid in biostimulants, we used quantitative spectrodensitometric thin-layer chromatography (TLC) (Negretskii, 1988; Savinskiy et al., 1991; Zizková et al., 2017), while gibberellins were measured by the spectrophotometric method (Muromtsev and Agnistikova, 1984; Waadt et al., 2015). The analysis of steroid compounds was carried out by gas chromatographic mass spectrometry on the device 6890N/5973inert (Agilent Technologies, USA) (Kamthan et al., 2012).

Working only with virus-free lines of plants, for the first set of experiments, seeds of wheat *Triticum aestivum* L. cv. Zimoyarka were surface sterilized successively in 1% KMnO_4_ solution (3 mins), 1% AgNO_3_ solution (2 mins), and 96% ethanol solution (1 min), after which they were thrice washed in distilled water. With the aim of stimulating seed germination and growth of wheat seedlings, as well as improving resistance to nematodes in the laboratory conditions, we have used the following physiologically optimal concentrations of microbial biostimulants

**Table:** 

Biostimulant	Concentration in distilled water, μL/L
Avercom	0.05
Avercom nova-2	0.05
Phytovit	2.5
Violar	1.3

For lower concentrations, the positive effect was reduced, while concentrations above 3 μL/L resulted in inhibition of seed germination and growth of wheat seedlings, as well as reduced resistance to nematode infestation. The seeds were germinated for 3 days in darkness at temperature 23°C in perlite-filled cuvettes, each containing 20 seeds and either distilled water or a solution of a biostimulant Avercom, Avercom nova-2, Phytovit, and Violar (Tsygankova et al., 2016). Upon germination, seedlings were grown for the next 3 weeks at 22–24°C under a 16/8 h photoperiod (light intensity 3000 lux) with relative humidity 60–80% in perlite-filled cuvettes. The artificial nematode-infested background was created by adding second stage larvae of the *H. avenae* nematode directly to perlite-filled cuvettes at concentration of 3–5 larvae per 1 mL of suspension at a rate of 5 mL per each wheat plant seed (i.e., 15–25 nematode larvae per each wheat plant seed). Additionally, leaf and stem surfaces of wheat seedlings were inoculated with suspension of nematode *H. avenae* larvae once per week at weekly intervals. Nematode larvae were obtained from nematode eggs using the method described in detail in our earlier published work (Tsygankova et al., 2012a).

The infection in our experiment occurred from the very start, due to the seeds growing on invasive background. As one of the parameters characterizing the effects of biostimulants on plants, we measured plant viability. With several different approaches being proposed over the years to define and measure viability of seeds (Scharpf, 1970; Rao et al., 2006; Shaban, 2013) and plants (Stergios and Howell, 1973; von Fircks and Verwijst, 1993), in our experiments we measured viability by planting 75 seeds in each trial, and then determining the percentage of surviving wheat plant seedlings at the end of the 3-week observation period. In terms of identifying the effects of biostimulants on plant growth, development, and protection against nematodes, we measured the percentage reduction in the overall level of nematode infestation on the surface of roots, stems, and leaves for experimental wheat seedlings grown on the invasive background in perlite filled cuvettes with biostimulants applied *in planta*, compared to control wheat seedlings to which no biostimulants were applied. The measurements were performed at the end of the 3-week period from the time the plant seeds were sown to the perlite-filled cuvettes with nematode larvae.

### 2.2. Isolation of Cytoplasmic mRNA and si/miRNA

To verify that biostimulants do indeed promote the additional production of plant si/miRNA complementary to either plant mRNA or nematode mRNA, we extracted total cytoplasmic RNA from plant cells using standard methods (Aviv and Leder, 1972; Davis et al., 1986; Tsygankova et al., 1998). The polymericity of isolated total RNA preparations was analyzed by electrophoresis in a 1.5% agarose gel stained with ethidium bromide in the presence of 7M urea by the Locker method (Locker, 1979). Separation of cytoplasmic poly(A)^+^mRNA (that is, mRNA) and poly(A)^−^mRNA was carried out by chromatography on oligo(dT) cellulose column (Aviv and Leder, 1972; Sambrook et al., 1989), with the purity of isolated cytoplasmic poly(A)^+^mRNA being analyzed using the Northern-blot method (Sambrook et al., 1989). Plant cytoplasmic si/miRNA was isolated from cytoplasmic RNA using the method we had developed earlier to analyse RNAi in rapeseed (Tsygankova et al., 2012a), and which was subsequently used to study RNAi in sugar beet (Tsygankova et al., 2012b), cucumber and potato (Tsygankova et al., 2014b), and Chinese cabbage (Biliavska et al., 2016c). We then performed dot blot hybridization (Sambrook et al., 1989) between this plant cytoplasmic si/miRNA and cytoplasmic mRNA isolated either from plant cells, or from nematode larvae cells. For the purpose of dot blot hybridization experiments, before isolation from the total plant cytoplasmic RNA population, si/miRNA were labeled *in vivo* with [^33^P] using Na_2_HP^33^O_4_ (Tsygankova et al., 2012a, 2014a,b). On the other hand, for experiments on inhibition of protein expression in a wheat germ cell-free system of protein synthesis (Marcus et al., 1974; Sambrook et al., 1989; Tuschl et al., 1999; Spirin and Swartz, 2008; Takai et al., 2010; Tsygankova et al., 2010), the original unlabeled si/miRNA were used (Tsygankova et al., 2014a,b). Purity of isolated si/miRNAs (i.e., the fact that their sizes were 21–25nt) was verified through electrophoresis in a 15% polyacrylamide gel (GE Healthcare Amersham, UK), which was stained with ethidium bromide solution and photographed under UV light (Sambrook et al., 1989). Once the gel was vacuum dried in a thermal gel dryer (LKB, Sweden), a fluorescent agent 2,5-diphenyl-1,3-oxazole (Abakumov et al., 1980) was added, and the gel was exposed for 2 months to X-ray film at -70°C for gel fluorography (Bonner and Laskey, 1974). Total cytoplasmic mRNA from cells of cereal cyst nematode *H. avenae* was isolated from nematode larvae using the methodology discussed in Tsygankova et al. (2012a).

### 2.3. Analysis of Synthesis of si/miRNAs Within Plant Cells and Their Silencing Activity

We studied the impact of biostimulants on production within plant cells of si/miRNA that target both plant mRNA associated with plant host genes, whose expression is upregulated during nematode infestation, thus promoting penetration of nematodes into plant roots (Safari et al., 2005; Tsygankova et al., 2012a, 2014a; Kong et al., 2015; Ali et al., 2017a; Chen et al., 2017), and these can be used as target genes for silencing (Koch and Kogel, 2014), and nematode mRNA associated with genes that control nematode life cycle, nematode housekeeping genes, nematode parasitism, or effector genes that are expressed during nematode entrance into plant cells (Tsygankova et al., 2012a, 2014a; Li et al., 2014; Chen et al., 2015, 2017; Xu et al., 2016; Ali et al., 2017b; Yang et al., 2017; Cui et al., 2018). For this purpose we perform dot blot hybridization of [^33^P]-labeled si/miRNA isolated from experimental plants with mRNA isolated from control plants, i.e., plants uninfested by nematodes and not treated by biostimulants, as well as with mRNA molecules isolated from nematode larvae. si/miRNA is isolated from nematode-infested plants grown in perlite-filled cuvettes from seeds treated by biostimulants *in planta*. To avoid potential losses of nucleic acids, hybridization was performed on Millipore AP-15 glass fiber filters composed of APT paper Kahl (2015). The filters were then dried, and the radioactivity of hybrid molecules per 20 μg±SD of mRNA was determined in a Beckman LS-100C counter using a toluene-based scintillation fluid (Tishler and Epstein, 1968; Bonner and Laskey, 1974). The level of hybridization provides a quantitative characteristic of the amount of *in planta* synthesized si/miRNA that is complementary with either plant mRNA, or nematode mRNA. To compare the effects of different biostimulants, levels of hybridization between plant si/miRNA and plant own mRNA were compared to baseline obtained for control plants uninfested by nematode and grown without the use of biostimulants.

To study silencing (inhibitory) activity of plant-derived si/miRNA, i.e., si/miRNA-mediated effects on translation of nematode mRNA, we used a wheat germ cell-free system of protein synthesis, which, together with other heterologous cell-free systems, is widely used to analyse the expression of proteins from viruses (Roberts and Paterson, 1973; Fogeron, 2015), plants (Takai et al., 2010; Harbers, 2014; Mmeka et al., 2014), mammals (Wakiyama et al., 2007; Singh et al., 2012), various prokaryotic and eukaryotic microorganisms (Guild et al., 2011; Harbers, 2014), and nematodes (El-Ansary and Al-Daihan, 2005; Wallace et al., 2010). It has been suggested that the wheat germ and other heterologous cell-free systems can also be used for studying the RNAi machinery or validating siRNAs for various organisms (Tuschl et al., 1999; Katzen et al., 2005; Mathonnet et al., 2007; Shyu et al., 2008). To improve the performance, the standard wheat germ extract system (Roberts and Paterson, 1973) that contains various cellular components essential for protein synthesis, was modified to include an energy generating system based on phosphocreatine kinase and phosphocreatine, magnesium acetate (Roy et al., 2011; Agalarov et al., 2014) and potassium acetate Wolin and Walter (1988) were added for improved translation, while additional stabilization was provided by adding spermidine (Spirin and Swartz, 2008). For the purpose of studying silencing (inhibitory) activity of si/miRNA, we used unlabeled si/miRNA obtained before their isolation from root and shoot tissues of wheat plants. Silencing activity was determined using the index of decreasing of incorporation [^35^S]-methionine into proteins (Sambrook et al., 1989; Titus, 1991; Spirin and Swartz, 2008). This index was measured as the level of radioactivity of polypeptides (in imp./count per min/1mg of proteins) obtained on a Millipore AP-15 glass fiber filter in toluene scintillator in the Beckman LS 100C scintillation counter (Bonner and Laskey, 1974; Osterman, 1981). The index of silencing activity of si/miRNA (in %) was then computed as a difference in the index of radioactivity of polypeptides synthesized on the template of nematode mRNA, obtained using si/miRNA isolated from experimental plants grown under application of biostimulants *in planta*, as compared to the same index obtained using si/miRNA isolated from control plants grown without biostimulants.

All experiments were performed in triplicate, and statistical analysis of data was performed using standard methods as described in Bang et al. (2010).

## 3. Results

**Figure 1:**
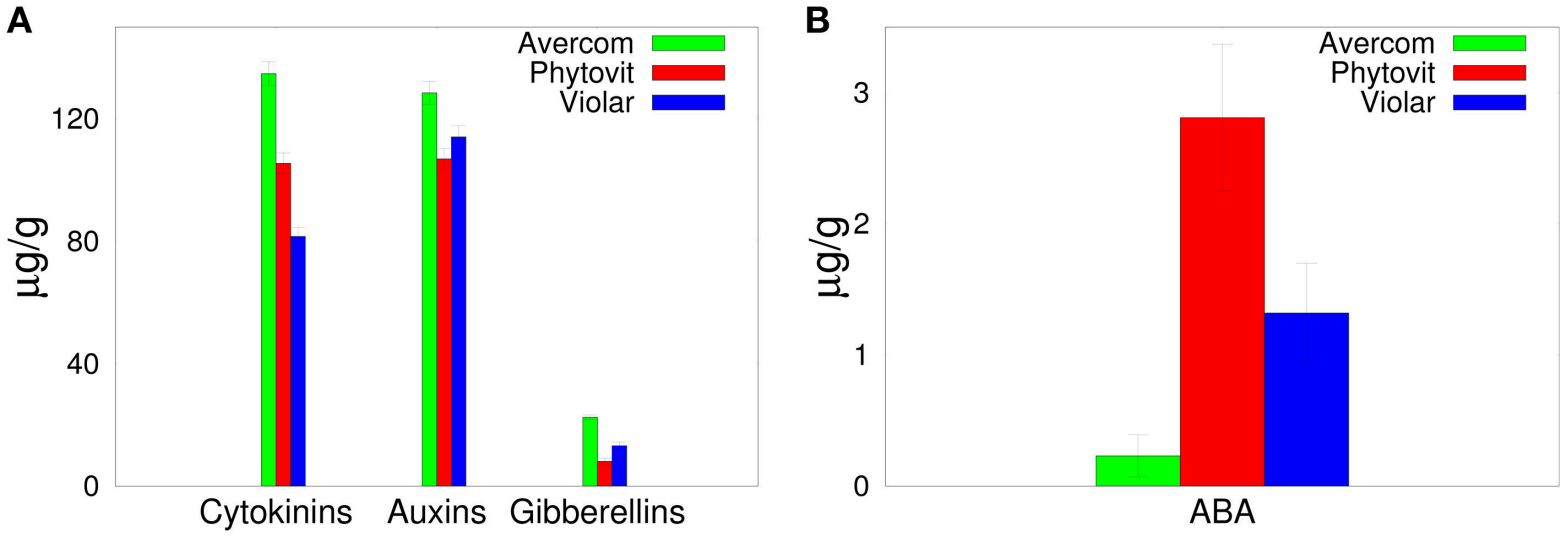
Plant hormone composition of biostimulants.

In order to understand the effects on biostimulants on plant growth and development, in Figure 1 we show plant hormone content of different biostimulants, including both auxins, cytokinins, gibberellins, and anti-stress (abscisic acid, ABA) hormones. It is worth noting that unlike stimulatory plant hormones, the inhibitory hormone ABA is present in biostimulants at a much lower concentration. All auxins in biostimulants are represented by indole-3-acetic acid (IAA) and indole-3-butyric acid and their derivatives.

**Figure 2:**
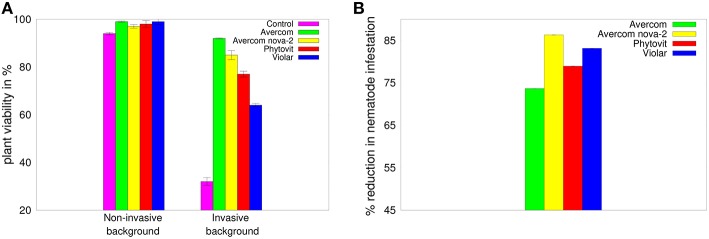
**(A)** Effects of biostimulants on viability of plants grown on non-invasive or invasive background in perlite-filled cuvettes from seeds treated *in planta* by biostimulants, as compared to a viability of control plants grown in perlite-filled cuvettes without biostimulants. **(B)** Reduction in infestation with *H. avenae* larvae of plants grown in perlite-filled cuvettes from seeds treated *in planta* by biostimulants, as compared to control plants grown in perlite-filled cuvettes in a nematode-infested background without biostimulants. The error bars in plot **(B)** are not visible, since they are almost 3 orders of magnitude smaller than the reported values.

As a first step in our experimental studies, we look at the effects of biostimulants on viability of 3-week-old wheat plants, as well as on their ability to withstand nematode infestation. Figure 2A shows that all biostimulants have a positive effect on plant viability in the absence of nematode infestation, but the differences between their influence on improving the viability of plants are very small. In contrast, adding invasive background reduces the viability of plants grown without biostimulants to 32%, while applying biostimulants *in planta* results in mitigating this effect and restoring plant viability to between 57 and 92%. Besides having a stimulatory effect on plant survivability in the presence of invasive background, biostimulants also appear to possess significant anti-nematode effect, as illustrated in Figure 2B. This Figure suggests that all biostimulants provide a significant anti-nematode effect, reducing the level of nematode infestation by 73–83% compared to plants grown without biostimulants.

Now that it has been established that biostimulants do have an anti-nematode effect, we look into whether this can be attributed to wheat plants producing additional si/miRNA complementary to mRNA of infested plants that is associated with plant genes, whose expression is upregulated during the entry of nematodes into plant cells, thus facilitating infestation of plants with nematodes, or to nematode mRNA associated with nematode genes that control nematode life cycle, nematode housekeeping genes, nematode parasitism or effector genes that are expressed during nematode entry into plant cells (Tsygankova et al., 2012a, 2014b; Li et al., 2014; Chen et al., 2015, 2017; Xu et al., 2016; Ali et al., 2017b; Yang et al., 2017; Cui et al., 2018).

**Figure 3:**
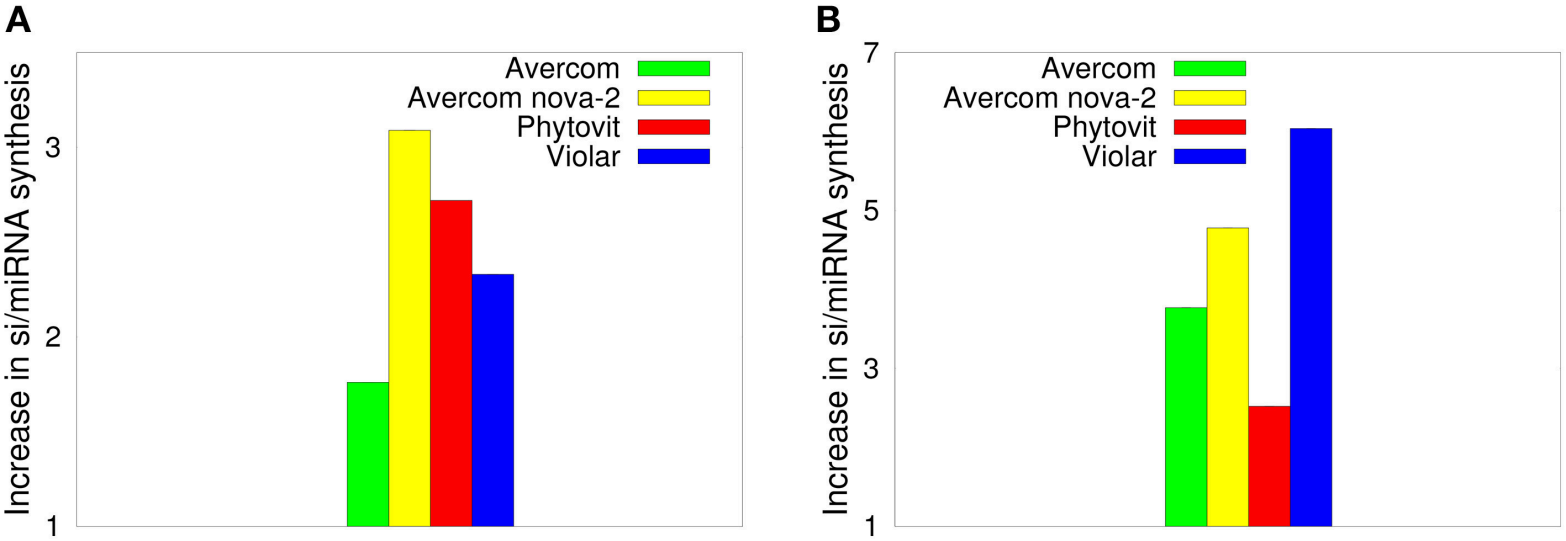
Effects of biostimulants on increasing the synthesis of si/miRNA in nematode-infested plants grown using biostimulants *in planta* that are complementary with mRNA from control plants **(A)**, and with nematode mRNA **(B)**, scaled by the amount of si/miRNA isolated from nematode-infested plants grown under the same conditions but without the use of biostimulants. The error bars are not visible, since they are three orders of magnitude smaller than the reported values.

The results of the dot blot hybridization between the si/miRNA isolated from plant cells grown without and with biostimulants with the mRNA extracted from either control plants, i.e., plants uninfested by nematodes and not treated by biostimulants, or from nematode larvae, are presented, respectively, in Figures 3A,B. In the case of hybridization with the plant mRNA, as shown in Figure 3A, we observe that biostimulants enhance the synthesis of si/miRNA complementary with plant mRNA by a factor of 1.7−3.09. Similar result is observed in Figure 3B for the experiment on dot blot hybridization between si/miRNA from nematode-infested plants and nematode mRNA, namely, application of biostimulants results in a significant enhancement of synthesis of si/miRNA complementary with nematode mRNA compared to plants grown from seeds without any biostimulants (Tsygankova et al., 2016). Interestingly, for Avercom and Avercom nova-2, the level of enhancement of synthesis of si/miRNA complementary with nematode mRNA is higher than analogous levels for si/miRNA homologous with plant mRNA, while for Phytovit the level of synthesis of si/miRNA complementary with nematode mRNA is smaller than the respective level for plant mRNA. Comparison with Figure 2 indicates that while the synthesis of complementary si/miRNA can be enhanced very significantly, the difference between levels of reduction in nematode infestation is much smaller, suggesting that out of the total si/miRNA produced in the plant, only a part of it contained in the feeding cells formed on plant roots can enter nematode through ingestion using a stylet (Maule et al., 2011; Tsygankova et al., 2012a; Cui et al., 2018), possibly resulting in the subsequent nematode death.

**Figure 4:**
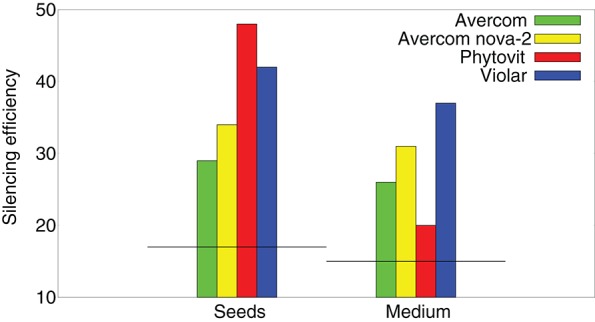
Effects of biostimulants on silencing activity of si/miRNA isolated from nematode-infested plants grown using biostimulants *in planta*, i.e., a percentage of inhibition of protein synthesis on the template of nematode mRNA in a wheat germ cell-free system, measured per 1μg of protein. Horizontal black lines denote the respective levels of silencing activity achieved by si/miRNA isolated from nematode-infested plants grown without the use of biostimulants.

To better understand how effective are the si/miRNA at suppressing nematode activity and causing their mortality, in Figure 4 we compare silencing activity of si/miRNA isolated from the plants grown without or with biostimulants, as measured in a wheat germ cell-free system. One observes a significant increase in silencing activity of si/miRNA on reducing the translation of proteins on the template of nematode mRNA (Tsygankova et al., 2016, 2019). Comparison of the results on silencing efficiency with Figure 3B suggests that it is not so much the actual amount of the additional si/miRNA produced in plant cells, but rather its specificity as determined by the level of silencing activity on the template of nematode mRNA that determines the degree to which biostimulants are effective in reducing nematode infestation.

These results show that biostimulants do indeed result in the production within wheat plants of si/miRNA complementary with nematode mRNA, and we have also observed a significant reduction in the overall protein production, as well as a marked increase in nematode mortality. Despite recent identification of some candidate genetic targets in *H. avenae* (Kumar et al., 2014; Cui et al., 2018), so far practical efforts have focused mostly on demonstrating the feasibility of RNAi by soaking nematode larvae either in dsRNA or gene-specific siRNA (Zheng et al., 2015; Liu et al., 2016), while plant-induced RNA silencing has remained elusive due to a significant variation in RNAi responses and poor understanding of the precise mechanisms of how RNAi products are transferred from plants into *H. avenae* (Maule et al., 2011; Lilley et al., 2012; Ali et al., 2019). In the absence of comprehensive understanding of genetic targets and the mechanisms of RNAi transfer between plant and nematode, we hypothesize that the observed reduction in the level of nematode infestation can be attributed to one of the genes, that are targeted by plant-produced RNAi products, being directly involved in protein synthesis, so silencing that particular gene results in the reduction of the overall protein level. Another possibility is that among the genes, whose expression is affected by the plant-produced sRNAs, there is one or several genes, whose silencing directly results in nematode death. With the recent work on splicing (Mackereth et al., 2011; Yang et al., 2016) demonstrating the possibility of multiple protein isoforms being encoded by the same genes, identifying the precise matching between sRNAs, produced in wheat plants under the influence of biostimulants, and specific *H. avenae* genes remains a challenging open problem.

## 4. Discussion

In this paper we have demonstrated the feasibility of using poly-component biostimulants derived from metabolites of various soil streptomycetes for protecting wheat plants against the cereal cyst nematode, which is known to be one of the main wheat parasites. Based on the guidance provided by theoretical models regarding how various chemical compounds are taken up by the plants through the seeds, we have subsequently performed experiments with biostimulants to investigate their efficiency in terms of plant uptake and subsequent nematicidal action.

Analysis of dot blot hybridization between the population of cytoplasmic si/miRNA from plants with mRNA from plants and nematodes suggests that application of biostimulants to germinating seeds results in the higher level of synthesis of si/miRNA in plant cells. These additional si/miRNAs are complementary to sequences present in the plant genes whose upregulated expression facilitates infestation of plants with nematodes, as well as to evolutionarily conserved sequences present in the nematode genes that control nematode life cycle, nematode housekeeping genes, nematode parasitism, or effector genes, and which are expressed during nematode entrance into plant cells (Tsygankova et al., 2012a, 2014a; Li et al., 2014; Chen et al., 2015, 2017; Xu et al., 2016; Yang et al., 2017; Cui et al., 2018). On the other hand, wheat germ cell-free experiments on silencing activity provide further evidence that biostimulants enhance the production in plant cells of si/miRNA that are capable of inhibiting (silencing) the translation of nematode mRNA having homologous evolutionarily conserved sequences, thus reducing the level of nematode infestation and improving plant resistance to nematodes. Taken together, these experiments demonstrate that as a method of nematode control, plant-delivered RNA interference is a complex process affected by a number of factors that include the mode of application of biostimulant, within-cell RNAi dynamics, specific properties of plant growth and development, as well as the uptake by nematodes and subsequent within-nematode dynamics. Although our results clearly indicate that streptomycete-derived biostimulants are indeed able to significantly reduce nematode infestation through plant-induced nematode mortality, the specific mechanism of genetic interactions between plant-produced RNAs and nematode mRNAs remains to be explored. At the same time, from a practical perspective these natural biostimulants do provide effective and safe means of nematode control for major staple crops.

There are several directions in which the work presented in this paper could be extended. Developing better theoretical models for uptake of biostimulants by seeds during their germination, as well as understanding more specific details of the transfer of biostimulants from the soil into plant roots, and the transfer of sRNAs from plants into nematode would provide important practical insights for optimizing the performance of biostimulants. Our future work will focus on identifying specific genes in *H. avenae* that are silenced by si/miRNAs produced in wheat plants under the action of biostimulants, which would provide a more targeted and effective approach to improving the performance of biostimulants as a means of nematode control.

## Author Contributions

KB developed theoretical models, and together with FF prepared a summary of theoretical results. VT, GI, and YB designed the experiments. VT, LB, GI, AY, and YB performed the experiments. The manuscript was written by KB and FF, and edited by all authors. All authors read and approved the final manuscript.

### Conflict of Interest Statement

The authors declare that the research was conducted in the absence of any commercial or financial relationships that could be construed as a potential conflict of interest.
